# Dynamics of Parkinson’s Disease Multimodal Complex Treatment in Germany from 2010–2016: Patient Characteristics, Access to Treatment, and Formation of Regional Centers

**DOI:** 10.3390/cells8020151

**Published:** 2019-02-11

**Authors:** Daniel Richter, Dirk Bartig, Siegfried Muhlack, Elke Hartelt, Raphael Scherbaum, Aristeides H. Katsanos, Thomas Müller, Wolfgang Jost, Georg Ebersbach, Ralf Gold, Christos Krogias, Lars Tönges

**Affiliations:** 1 Department of Neurology, St. Josef-Hospital, Ruhr-University Bochum, 44801 Bochum, Germany; daniel.richter-c34@rub.de (D.R.); siegfried.muhlack@rub.de (S.M.); elke.hartelt@rub.de (E.H.); raphael.scherbaum@rub.de (R.S.); ar.katsanos@gmail.com (A.H.K.); ralf.gold@rub.de (R.G.); christos.krogias@rub.de (C.K.); 2 DRG MARKET, D-49069 Osnabrück, Germany; dirk.bartig@drg-market.de; 3 Second Department of Neurology, National and Kapodistrian University of Athens, Athens 15771, Greece; 4 Department of Neurology, St. Joseph Krankenhaus Berlin-Weißensee, 13088 Berlin, Germany; Th.Mueller@alexianer.de; 5 Center for Movement Disorders, Parkinson-Klinik Ortenau, 77709 Wolfach, Germany; w.jost@parkinson-klinik.de; 6 Department of Neurology, University Hospital Freiburg, 79104 Freiburg, Germany; 7 Neurologisches Fachkrankenhauses für Bewegungsstörungen/Parkinson, Kliniken Beelitz, 14547 Beelitz, Germany; Ebersbach@kliniken-beelitz.de; 8 Neurodegeneration Research, Protein Research Unit Ruhr (PURE), Ruhr University Bochum, 44801 Bochum, Germany

**Keywords:** Parkinson disease, multiprofessional therapy, inpatient treatment, multimodal complex treatment

## Abstract

Parkinson’s disease (PD) is currently the world’s fastest-growing neurological disorder. It is characterized by motor and non-motor symptoms which progressively lead to significant clinical impairment, causing a high burden of disease. In addition to pharmacological therapies, various non-pharmacological treatment options are available. A well established and frequently used multiprofessional inpatient treatment concept in Germany is “Parkinson’s disease multimodal complex treatment” (PD-MCT) which involves physiotherapists, occupational therapists, speech therapists, and other specializations for the optimization of treatment in PD (ICD G20) and other Parkinsonian syndromes (ICD G21 and G23). In this study we analyze the PD-MCT characteristics of 55,141 PD inpatients who have been integrated into this therapy concept in Germany in the years 2010–2016. We demonstrate that PD-MCT is increasingly applied over this time period. Predominately, PD patients with advanced disease stage and motor fluctuations in age groups between 45 and 69 years were hospitalized. In terms of gender, more male than female patients were treated. PD-MCT is provided primarily in specialized hospitals with high patient numbers but a minor part of all therapies is performed in a rather large number of hospitals with each one treating only a few patients. Access to PD-MCT differs widely across regions, leading to significant migration of patients from underserved areas to PD-MCT centers–a development that should be considered when implementing such therapies in other countries. Furthermore, our data imply that despite the overall increase in PD-MCT treatments during the observational period, the restricted treatment accessibility may not adequately satisfy current patient’s need.

## 1. Introduction

Parkinson disease (PD) is currently the world’s fastest-growing neurological disorder [1]. It is characterized by motor symptoms such as bradykinesia, tremor, muscular rigidity or gait instability and is classified as primary Parkinson syndrome in the German DRG system [2]. Other clinically distinct Parkinsonian syndromes like vascular Parkinsonism or progressive supranuclear palsy (PSP) are subcategorized as secondary Parkinson syndromes, or other degenerative diseases of the basal ganglia. All Parkinsonian syndromes frequently comprise non-motor symptoms such as depression, pain, and sleep disorders which substantially decrease the quality of life of PD patients [3]. In Germany, the prevalence of PD is estimated in at least 180,000 to 220,000 patients [4,5,6]. It is expected that in 2040 more than 14 million people will suffer from PD, underlining the great impact of this disease on the health system now and in the future [7]. In order to improve early diagnosis and optimal therapy initiation in Parkinson’s patients, the widest possible availability of specific diagnostics and individualized treatment in a multiprofessional team is desirable [8,9].

In Germany, there exist specialized inpatient units for patients with PD and other Parkinsonian syndromes which perform a so-called Parkinson disease multimodal complex treatment (PD-MCT) in a multiprofessional setting. Prerequisite for the reimbursement of health insurance are the documented physician expertise for PD, a constant and careful anti-parkinsonian drug titration as well as the application of activating therapies with a duration of at least 7.5 h per week. The basis for PD treatment is the multidisciplinary team which involves different professions such as physiotherapists, occupational therapists, and speech therapists as well as other paramedical disciplines. Inpatient treatment is generally applied from 7 days up to a total of 21 days, but mostly a therapy duration of about 14 days is chosen [10]. The effectiveness of PD-MCT for the clinical improvement of motor and non-motor function has been examined and demonstrated previously [11,12,13]. Especially in advanced stages of PD, this therapy concept is often needed [14].

In a recent study on PD patients who received general inpatient treatment in Germany, we saw strong momentum in 2010–2015 with patient numbers rising. Patients with motor fluctuations were especially in need of treatment. However, the treatment approaches for these patients and applied therapies have not been analyzed. In addition, there is very little information on the distribution of specialized treatment facilities or centers in Germany and whether there are regions that suffer from restricted accessibility to treatment. When examining treatment modalities for outpatients in Germany, recent data show that there are dramatic regional differences in diagnostic and therapeutic workups of PD [8]. Importantly, a recent analysis has found a substantial increase in PD prevalence and an increased annual healthcare utilization in Germany [15].

In order to evaluate the use and accessibility of stationary multiprofessional PD-MCT for PD inpatients in Germany we provide for the first time a thorough analysis about the patient characteristics and dynamics of PD-MCT application in Germany for the years 2010–2016 based on G-DRG statistics (“diagnosis-related groups”) and structured quality reports in 55,141 PD patient cases. We recognize important regional differences in terms of PD-MCT rates and their availability, which may serve as a basis for further planning of the availability of these resources in Germany.

## 2. Materials and Methods

Analyses reflecting the extent and type of Parkinson’s inpatient treatment were based on the statistical evaluation of the German Diagnosis-Related Groups (G-DRG) data from 2010 to 2016 (DRG-statistic, Federal Statistical Office, www.destatis.de) as well as the mandatory structured quality reports of hospitals for the reporting year 2016 (according to §137, 3.1 No.4; Social Code Book V of Germany: the quality report of the hospitals is used only partially or in extracts. A complete unchanged version of the quality reports can be found at www.g-ba.de). For financial compensation in Germany, all inpatient cases are encoded by International Statistical Classification of Diseases and Related Health Problems 10th revision, German modification (ICD-10-GM) and relevant operating and procedure keys (OPS codes). In the analyzed time period there was no change in the German coding system or a revision of the ICD version. The G-DRG data were used for all calculation based on the place of patients’ residence, whereas the data extracted from the structured quality reports reflects the place of patients’ treatment.

From DRG-statistic and structured quality reports we extracted all cases with the main diagnoses ICD-10 codes G20.-, G21.- and G23.- (Table 1), as well as all cases with associated OPS 8-97d.- (PD-MCT, Table 2). We calculated mean age, gender, and treatment rates for main diagnoses and PD-MCT procedures.

To assess for potential disparities between the predefined age groups, we plotted the relevant percentages and 95% confidence intervals (95% CI) for all outcomes of interest and for each consecutive year, stratified by the age group. Pooled overall estimates and age group estimates were calculated using the random-effects model. Given the expected heterogeneity both within and between groups, we also provided the corresponding 95% prediction intervals (95% PI) to allow for a better appreciation of the uncertainty around cumulative estimates. To evaluate for potential disparities with regard to existing fluctuations, we estimated odds ratios (ORs) with the corresponding 95% CIs for all outcomes of interest. Cumulative estimates were again provided using the random effects model. Both within and between group differences in all analyses were assessed with the Cochran’s test for heterogeneity. Analyses were performed with the Stata Statistical Software Release 13 (StataCorp LP, College Station, TX, USA).

Regional analyses were done by data aggregation considering the 401 German administrative counties and cities. In order to determine the extent of patient migration, the specific figures for number of cases per 100,000 inhabitants and treatment rates were calculated for each county. Analyzing data from the structured quality reports of the hospitals, we calculated the number of main diagnosis G20–G23 and OPS 8–97d (PD-MCT) treated in each hospital. We stratified hospitals by number of main diagnoses and OPS 8–97d performed per year into the following categories: <3, 3–12, 13–52, 53–520, >520. These categories were chosen for the following reasons:

- Single cases of PD treatments and possible false entries (<3): no regular experience can be presumed.
- Occasional PD treatments (3–12 per year): with less than one treatment per month only occasional experience can be presumed.
- Regular PD treatments (13–52 per year): with up to 1 treatment per week, a regular experience can be presumed.
- Frequent PD treatments (53–520 per year): with 1 to 10 treatments per week, a good experience with a good regular standard can be presumed.
- High volume PD treatments (>520 per year): with more than 10 treatments per week, a very good experience and a high-performance standard can be presumed.

We stratified hospitals by number OPS 8-97d in the same way except for categories:

- Frequent PD-MCT (52–104 per year): with 1 to 2 treatments per week, a good standard with an experienced multiprofessional team can be presumed.
- High volume PD-MCT (>104 per year): with more than 2 treatments per week, a highly experienced multiprofessional team can be presumed.

## 3. Results

### 3.1. Inpatient Treatment and PD-MCT on Federal Level

#### 3.1.1. Case Numbers of Inpatient Treatment for PD and Other Basal Ganglia Disorders and Proportion of PD-MCT

**Figure 1:**
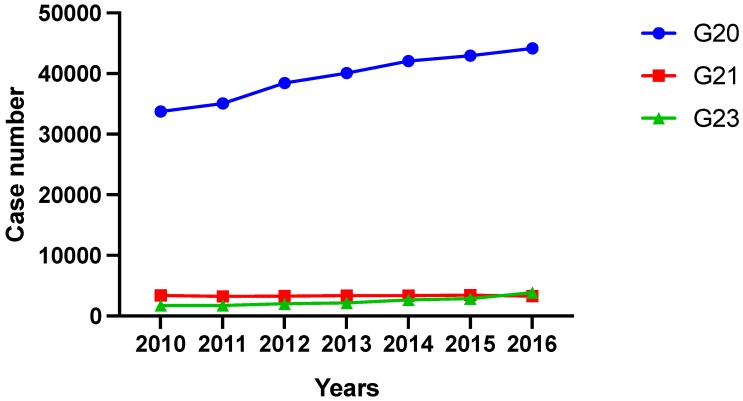
Case number development of inpatient treatment of PD and other basal ganglia disorders (ICD G20–G23) from 2010 to 2016 divided into ICD-categories. G20 = primary Parkinson’s syndrome; G21 = secondary Parkinson’s syndrome; G23 = other degenerative disease of the basal ganglia.

In 2010 a total number of 33,760 inpatient treatments were performed for primary Parkinson’s syndrome (G20). Treatment numbers increased up to 44,192 cases per year in 2016. Concerning secondary Parkinson’s syndromes (G21), treatment numbers decreased from 3388 in 2010 to 3271 cases in 2016. Case numbers of patients with other degenerative diseases of the basal ganglia (G23) more than doubled from 2010 (1749 cases) to 2016 (3858 cases), but represent only a minor part of all treatments (Figure 1).

**Figure 2:**
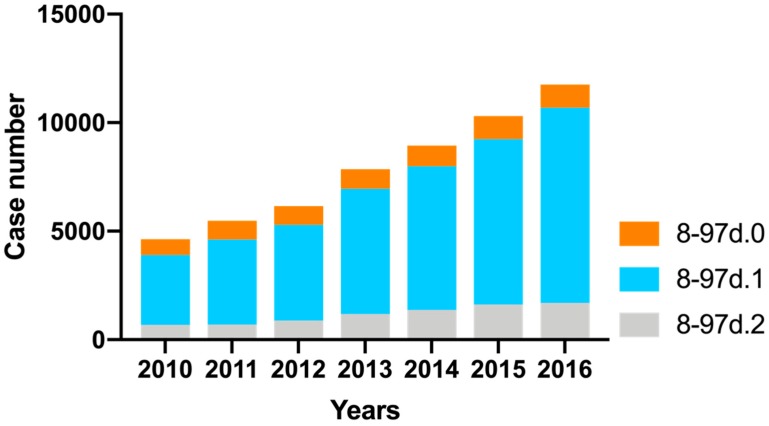
Case number development of PD-MCT from 2010 to 2016 divided into subcategories. 8–97d.0 = PD-MCT treatment of 7–13 days; 8–97d.1 = PD-MCT treatment of 14–20 days; 8–97d.2 = PD-MCT treatment of at least 21 days.

Of all patients describe above, a proportion received inpatient PD-MCT. PD-MCT case numbers steadily increased from the year 2010 with 4635 treatments up to 11,755 treatments in 2016 (absolute increase of 7120 cases or 153.6%). The largest increase was seen for OPS 8–97d.1 (14–20 treatment days; absolute: 5777 cases; relative: 179.5%) followed by OPS 8–97d.2 (at least 21 treatment days; absolute: 1016 cases; relative: 148.8%). In the OPS 8–97d.0 subgroup (7–13 treatment days) there was a minor increase (absolute: 327 cases; relative: 44.6%) (Figure 2).

#### 3.1.2. Age and Gender Characteristics and Treatment Rates of PD-MCT

The mean age of the patients participating in PD-MCT was similar over the years (2010: 72.4 years; in 2016: 72.8 years) (Table 3).

Differentiating PD-MCT treatment rate for gender, proportionately more male than female patients were included in 2010 (male to female ration of 57 vs 43 %). By 2016, the increase of treated cases was higher for men (160.7%) than for women (144.3%). This resulted in more pronounced male treatment emphasis (male to female ratio of 59 vs. 41%), which, however, reflects higher hospitalization for male PD patients in general (Table 4).

About 90% of all PD-MCT treatments conducted in 2016 were applied to patients with a primary Parkinson’s syndrome resulting in an overall treatment rate of 23.8% for this subgroup. Of all PD-MCT, another 10% were applied to patients with secondary Parkinson’s syndrome and to patients with other degenerative diseases of the basal ganglia. The overall percentage of all PD patients (G20+G21+G23) receiving PD-MCT treatment increased from 11.9% to 23.0% during the observational period (Table 4).

**Figure 3:**
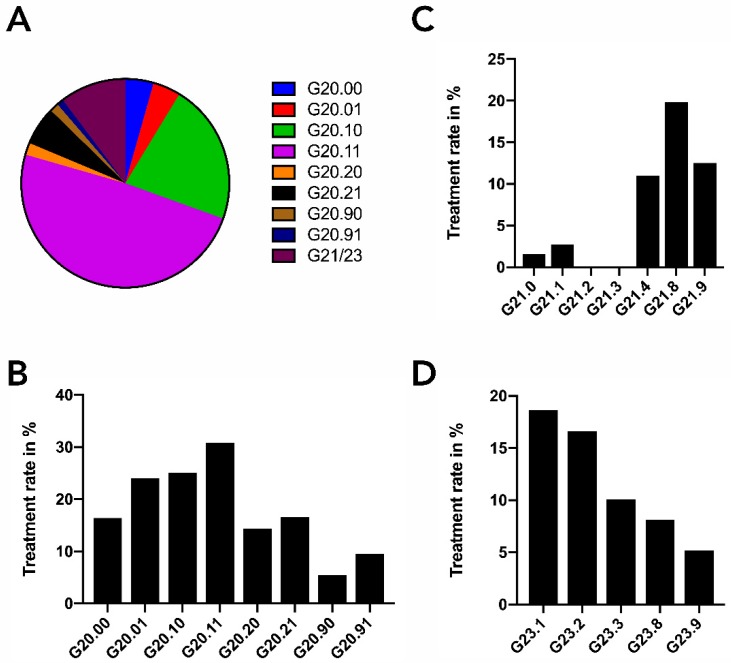
PD-MCT procedures of the year 2016. (**A**) Relative shares of all PD-MCT procedures subdivided for PD and other basal ganglia disorders (ICD G20–G23). ICD codes are described in detail in Table 1. (**B**–**D**) PD-MCT treatment rate in patients with primary Parkinson’s syndrome (ICD G20.–) (**B**), with secondary Parkinson’s syndrome (ICD G21.–) (**C**) or with other degenerative disease of the basal ganglia (ICD G23.–) (**D**).

Dividing patients with primary Parkinson’s syndrome (G20) into its subcategories, we found that PD-MCT was primarily applied to patients with moderate to severe impairment (G20.1–, 70.7%). Patients with no or only mild impairment (G20.0–, 8.7%) received less treatment, as well as the more advanced patients (G20.2–, 7.9%). Interestingly, in the G20.1 category, patients with motor fluctuations (G20.11) were twice as much treated (48.9% vs. 21.8%) as patients without motor fluctuations (G20.10). The share of all PD-MCT treatments conducted in patients with G21 or G23 diagnosis was about 10% (Figure 3A). Treatment rates for the different subcategories of Parkinson syndrome (G20, G21, G23) are shown in Figure 3B–D. The highest PD-MCT rate (30.8%) was found for PD patients with motor fluctuations and a moderate to severe impairment (G20.11).

**Figure 4:**
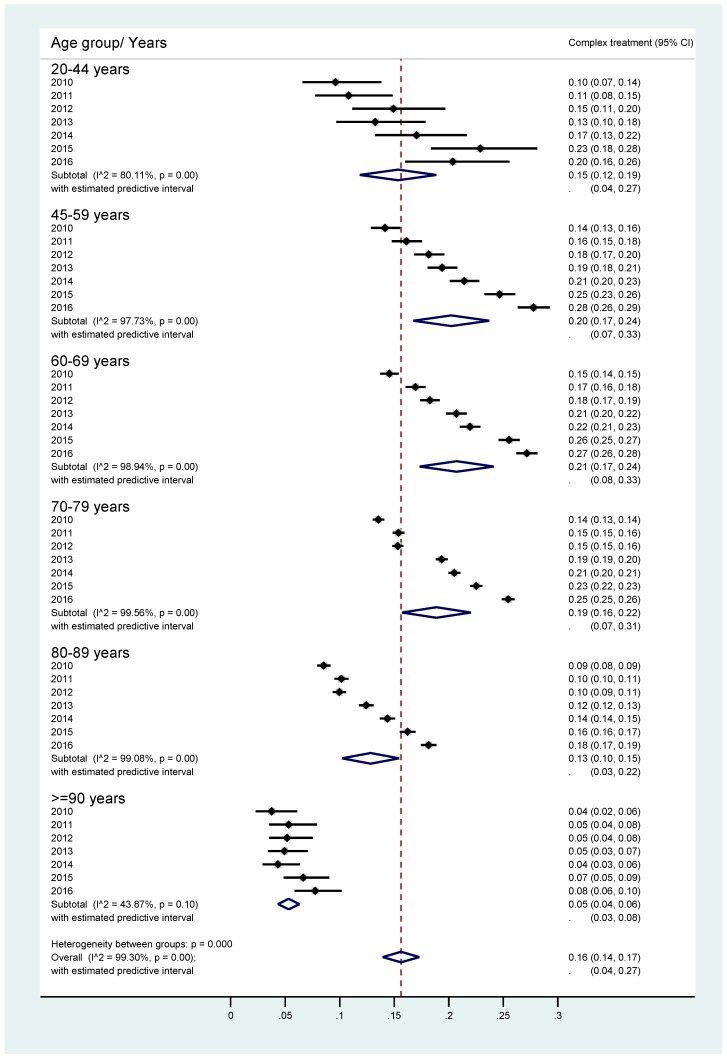
PD-MCT rates for primary Parkinson syndrome from 2010–2016 stratified by age group. Horizontal bars depict percentages and 95% confidence intervals (CI) to receive PD-MCT in relation to all stationary PD treatments in specified years for predefined age groups. Diamonds depict respective averages of the years 2010–2016 for predefined age groups and the overall average.

If patients with primary Parkinson’s syndrome (G20) were further stratified by age group, we found strongly increased PD-MCT rates between 2010 and 2016, especially for those aged 45–59 years and 60–69 years. Very old patients (≥ 90 years), but also early-onset PD patients (20–44 years) received less PD-MCT (Figure 4).

**Figure 5:**
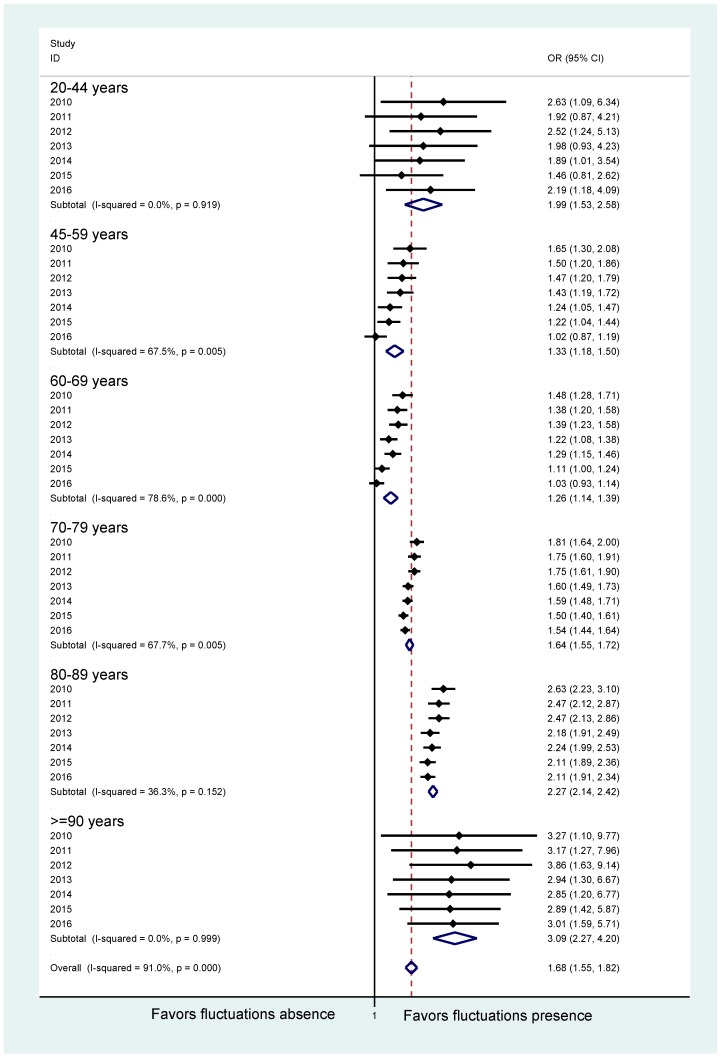
Odds ratio for primary Parkinson syndrome with or without motor fluctuations to receive PD-MCT between 2010 and 2016. Horizontal bars depict estimated odds ratios (ORs) with the corresponding 95% confidence intervals (CI) to receive PD-MCT depending on a diagnosis without (left) or with (right) motor fluctuations in specified years for predefined age groups. Diamonds depict respective averages of the years 2010–2016 for predefined age groups and the overall average.

Interestingly, the odds ratio to receive PD-MCT with the presence of motor fluctuations was significantly higher for all age groups. It was particularly likely to be subjected to this therapy if motor fluctuations were present in the young (20–44 years) or those of older age (80–89 years and ≥ 90 years) (Figure 5).

### 3.2. Inpatient Treatment and PD-MCT on Hospital Level

#### 3.2.1. General PD Inpatient Treatment

In 2016, 44,000 inpatients (ICD G20+G21+G23) were hospitalized in 1296 hospitals in Germany for general treatment of their disease. Importantly, about 18.7% of all patients were hospitalized in only seven hospitals. These hospitals treated more than 520 cases a year, which is a case load of more than 10 patients per week. Another large proportion of PD cases (56.4%) were treated in 223 hospitals, which handled between 53 and 520 patients per year corresponding to one to 10 cases per week. These indicate that 75.1% of all patients were treated in only 17.7% of all hospitals. The remaining 740 hospitals (57.1 % of all hospitals) treated only 1–12 patients per year (Table 5).

Interestingly, hospitalized PD patients were treated not only in neurology departments but also in other disciplines. In 2016, 77.0% of all inpatient treatments (for ICD G20+G21+G23) were conducted in a neurology department, but 12.0% were treated in a department of internal medicine and 7.2% in a department of geriatrics (Table 6).

#### 3.2.2. Inpatient PD-MCT

A total of 207 hospitals provided PD-MCT treatment for PD patients (ICD G20+G21+G23) in 2016 in Germany. 58.7% of all PD-MCT were conducted in only 26 specialized hospitals. This corresponds to a rate of more than two conducted PD-MCT treatments per week. The hospital with the highest PD-MCT treatment number in 2016 performed more than 6% of all PD-MCT treatments in Germany. 15.0% of all PD-MCT treatments were performed in 24 different hospitals with an average treatment number of one to two treatments per week. The remaining 26.3% of the PD-MCT treatments were performed in 157 hospitals that had a mean PD-MCT treatment rate of less than one treatment a week (Table 7).

Concerning treating disciplines, the highest number of PD-MCT was performed in departments of Neurology (94.1%), but 5.0% of the PD-MCT treatments were still conducted in departments of Internal Medicine (Table 8).

### 3.3. Regional Distribution of General PD Inpatient Treatment and PD-MCT in Germany

#### 3.3.1. General PD Inpatient Treatment

**Figure 6:**
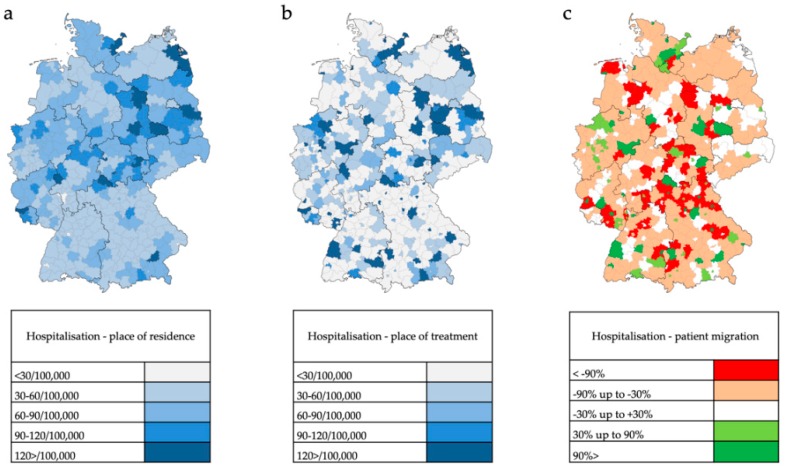
PD (G20+G21+G23) inpatient treatment in 401 administrative counties in Germany in the year 2016. (**a**) Crude rate of inpatient treatment per 100,000 inhabitants based on patients’ place of residence. (**b**) Crude rate of inpatient treatment per 100,000 inhabitants based on patients’ place of treatment. (**c**) Patient migration rate of inpatient PD treatment calculated as relative difference between patients’ place of residence and patients’ place of treatment. Values near to 0% indicate a balanced migration ratio. Values less than −30% indicate a distinct out-migration while values greater than +30% indicate a distinct immigration.

The crude rates (case number per 100,000 inhabitants) for general inpatient PD treatment according to the place of residence of PD patients in 401 administrative counties of Germany in 2016 are shown in Figure 6a. Relatively high treatment rates are found predominately in rural regions and in parts of eastern Germany. Concerning treatment rates based on patients’ place of treatment there is an even more heterogenous distribution with the formation of regional centers (Figure 6b). Resulting relative migratory movements of PD patients from their place of residence to treatment centers are depicted in Figure 6c.

#### 3.3.2. Inpatient PD-MCT

**Figure 7:**
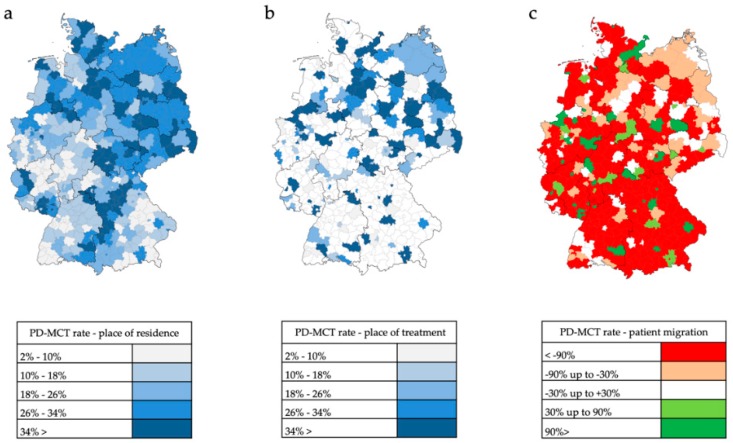
Inpatient PD-MCT in 401 administrative counties in Germany in the year 2016. (**a**) Ratio of PD-MCT to PD inpatients based on patients’ place of residence. (**b**) Ratio of PD-MCT to PD inpatients based on patients’ place of treatment. (**c**) Patient migration rate (G20+G21+G23) calculated as relative difference between patients’ place of residence and patients’ place of treatment of PD-MCT number. Values near to 0% indicate a balanced migration ratio. Values less than −30% indicate a distinct out-migration while values greater than +30% indicate a distinct immigration.

**Figure 8:**
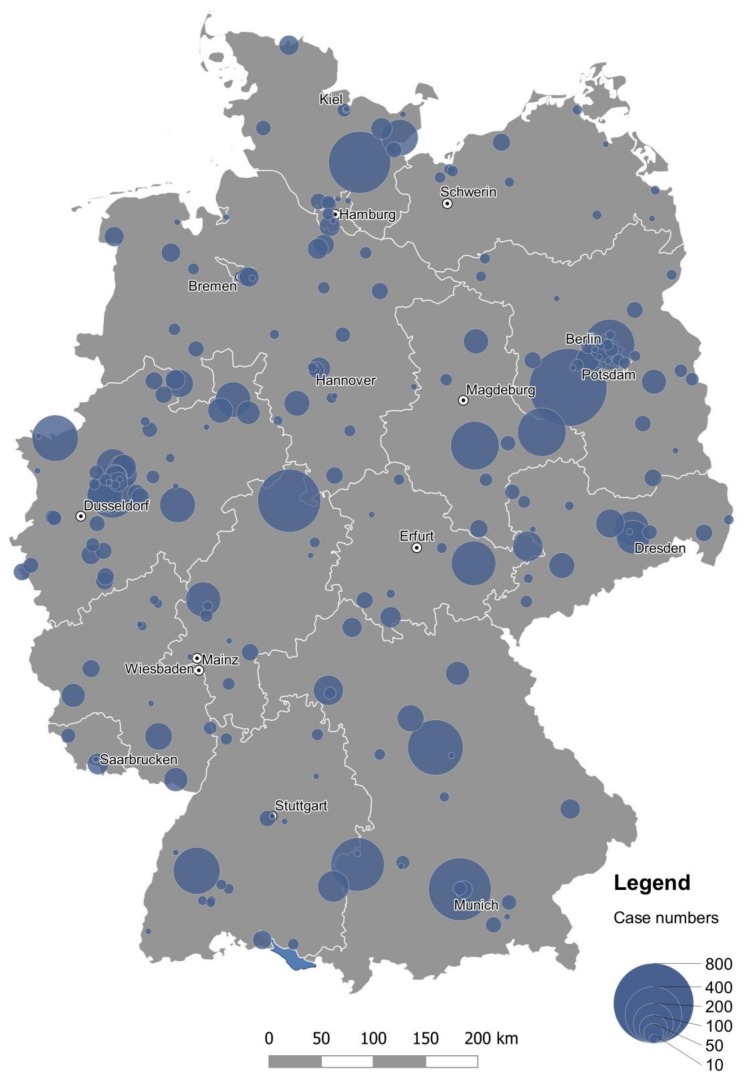
Area-proportional representation of PD-MCT case numbers in German hospitals in 2016.

Patients who had been hospitalized and undergone a PD-MCT again lived relatively often in rural areas or in eastern and southeastern Germany (Figure 7a). The place of PD-MCT was even more pronouncedly concentrated in regional centers (Figure 7b, Figure 8). Relative migratory movements of patients for PD-MCT from their place of residence to treatment centers are depicted in Figure 7c.

In 256 of 401 counties in Germany (63.8%), there is a non-balanced ratio between requirement and service offer for PD-MCT causing massive patient migration. The mean migratory movement rate of PD patients to receive PD-MCT was as high as 61% and was found in the northern, eastern and central south part of Germany. In western Germany, the migratory movement for PD-MCT treatment was less prominent.

## 4. Discussion

PD-MCT is a therapeutic concept which is increasingly used in Germany. In this manuscript, we described the characteristics of patients receiving this therapy and current trends in the use of this therapy. The ratio of inpatient hospitalization for PD-MCT in comparison to general PD therapy strongly differs depending on the Parkinson’s clinical phenotype and its intensity of administration does not automatically reflect treatment numbers of a general PD therapy. The availability of PD-MCT is highly dependent on specialized therapy centers, which leads to strong migration movements.

In Germany, treatment numbers of PD-MCT constantly rose from 2010 to 2016. This corresponds to the overall increasing hospitalizations for PD in Germany, as was found for a similar time period [2]. Of all PD patients, the rate of PD-MCT increased from 11.9% in 2010 to 23.0% in 2016, reflecting that now more patients are in need of an intensified multiprofessional therapy. In particular, patients with motor fluctuations in middle age groups were hospitalized because outpatient treatment of their condition may not have been sufficient or could not meet their therapeutic needs. The increasing application of PD-MCT is thus a positive observation and is an indicator of improved therapeutic care. The overall application of PD-MCT for all patients is still rather low with as much as 2% [15]. However, very much clinically advanced patients do not receive this treatment to a similar degree. Here, the inability to take part in the main therapeutic procedures may be a limiting factor.

There were many more PD-MCT for men than women for the treatments we studied. However, a less prominent female representation, and thus stronger male representation can be explained with the overall increased prevalence of male PD patients [16], which is also reflected in the overall hospitalizations in Germany [2].

Interestingly, more than 90% of PD-MCT is used in patients with primary Parkinson’s syndrome (G20) because this is the main patient group which presents in the clinics. In addition, other clinical syndromes such as secondary Parkinson’s syndromes (G21) or patients with other degenerative diseases of the basal ganglia (G23) are in need of a specialized PD-MCT. Because patient numbers of these categories are also rising [2] the therapeutic offers must adapt to the needs of these patients and provide e.g., more training for gait disturbances or instability, which is present in patients with progressive supranuclear palsy [17].

Regarding treatment duration, we have found the largest increase of PD-MCT treatment in the subcategory of 14–20 days, but also treatments with durations of 21 days and more were rising. In one monocentric study in Germany, inpatient treatment of 124 PD patients in a specialized PD unit using the PD-MCT treatment standard was evaluated. Several motor and non-motor scores were assessed before and after a therapy duration of 21 days with the result of a strong clinical benefit in this setting [11]. These data demonstrate that a treatment duration of 21 days is effective to optimize PD patient performance. Whether shorter treatment durations are similarly effective has to be shown in subsequent studies.

Treatment numbers of PD-MCT vary to a large extent between different hospitals. While a few specialized clinics initiate treatment of more than 2 cases per week, the vast majority of hospitals treats only one new case per week or even one new case per month. If the basic requirements of PD-MCT infrastructure are met, a hospital can perform and charge this procedure. However, there is still no generally accepted quality standard or even certification of centers that could guarantee state-of-the-art treatment.

Importantly, a large majority of the PD patients need to leave their residence counties to receive PD-MCT. Especially in rural areas and some parts of eastern and south eastern Germany, access to PD-MCT is limited and patients have to migrate to a large extent to other counties. It has to be discussed in the professional societies, by health authorities and of course by patients, if the current strong concentration on treatment centers is sufficient for overall treatment accessibility or if a more broadly available infrastructure with more regional centers should be provided, too. As the prevalence of PD is predicted to increase rapidly in the future [7], it seems to be worthwhile to now improve the treatment infrastructure so that patients in need can more easily access PD-MCT in Germany. That improvements of PD therapeutic concepts are also cost-efficient has recently been shown in a comprehensive retrospective analysis of medical claims data for Dutch patients [12].

## 5. Limitations

In this study we analyzed comprehensive data on the PD-MCT treatment for PD patients in Germany. These data are based on documented diagnoses and procedures in the G-DRG system, the correctness of which is regularly monitored by insurance companies. Some diagnoses or procedures may not be adequate in some cases but as up to 20% of all coded cases are controlled and corrected or will be degraded in payment if inadequately labelled, we assume a high degree of accuracy. Furthermore, we employ the data of 6 subsequent years, providing a robust data base for our analyses.

## 6. Conclusions

Parkinson’s disease prevalence increases worldwide, even in Germany. Patients in need of a specialized inpatient therapy can be transferred to PD-MCT in order to substantially improve their clinical symptoms both for motor and non-motor issues. This treatment option is increasingly applied, and various specialized centers provide this therapy to a substantial proportion of patients in Germany. For future planning, access to this treatment option should be further developed at local but also at regional or even national levels. A permanent scientific assessment of its effectiveness should be made.

## Figures and Tables

**Table 1:** Parkinson diagnoses and corresponding code of the International Statistical Classification of Diseases and Related Health Problems 10th revision, German modification (ICD-10-GM).

ICD-10	Diagnoses
**G20.–**	Primary Parkinson’s syndrome
**G20.00**	Primary Parkinson’s syndrome without or with less impairment and no fluctuation of action
**G20.01**	Primary Parkinson’s syndrome without or with less impairment and fluctuation of action
**G20.10**	Primary Parkinson’s syndrome moderate to severe impairment and no fluctuation of action
**G20.11**	Primary Parkinson’s syndrome moderate to severe impairment and fluctuation of action
**G20.20**	Primary Parkinson’s syndrome with the most serious impairment and no fluctuation of action
**G20.21**	Primary Parkinson’s syndrome with the most serious impairment and fluctuation of action
**G20.90**	Primary Parkinson’s syndrome not further defined and no fluctuation of action
**G20.91**	Primary Parkinson’s syndrome not further defined and fluctuation of action
**G21.–**	Secondary Parkinson’s syndrome
**G21.0**	Neuroleptic malignant syndrome
**G21.1**	Medication induced Parkinson’s syndrome
**G21.2**	Parkinson’s syndrome caused by other exogenic agents
**G21.3**	Post encephalitic Parkinson’s syndrome
**G21.4**	Vascular Parkinson’s syndrome
**G21.8**	Other secondary Parkinson’s syndrome
**G21.9**	Secondary Parkinson’s syndrome not further defined
**G23.–**	Other degenerative disease of the basal ganglia
**G23.0**	Neurodegeneration with Brain Iron Accumulation
**G23.1**	Steele–Richardson–Olzewksi syndrome
**G23.2**	Multi system atrophy
**G23.8**	Other specified degenerative disease of the basal ganglia
**G23.9**	Other degenerative disease of the basal ganglia not further defined

**Table 2:** Procedural codes for PD-MCT.

OPS-Code	Signification
8-97d	PD-MCT treatment of any duration
8-97d.0	PD-MCT treatment of 7–13 days
8-97d.1	PD-MCT treatment of 14–20 days
8-97d.2	PD-MCT treatment of at least 21 days

**Table 3:** Mean age of the PD-MCT-patients according to subcategories for the years 2010 and 2016.

OPS	2010	2016
8-97d.0	73.4	73.8
8-97d.1	72.1	72.6
8-97d.2	72.5	73.1
8-97d	72.4	72.8

**Table 4:** Data and treatment rates for PD-MCT separated into ICD subcategories.

	Inpatient Treatment [Male/Female]				PD-MCT [Male/Female]		PD-MCT Treatment Rate [Male/Female]
Year	G20 Cases	G21 Cases	G23 Cases	G20+G21+G23 Cases	8–97d Cases	8–97d Ratio	G20+G21+G23 Ratio
**2010**	19,111/14,649	1834/1554	901/848	21,846/17,051	2642/1993	57%/43%	12%/12%
**2011**	19,900/15,181	1779/1459	944/802	22,623/17,442	3075/2410	56%/44%	14%/14%
**2012**	22,257/16,208	1826/1460	1128/894	25,211/18,562	3596/2564	58%/42%	14%/14%
**2013**	23,509/16,588	1840/1499	1178/990	26,527/19,077	4642/3212	59%/41%	17%/17%
**2014**	24,936/17,137	1912/1458	1368/1290	28,216/19,885	5195/3754	58%/42%	18%/19%
**2015**	25,478/17,512	1957/1485	1571/1304	28,996/20,301	6037/4266	59%/41%	21%/21%
**2016**	26,399/17,793	1909/1362	2122/1736	30,430/20,891	6887/4868	59%/41%	23%/23%

**Table 5:** Number of cases per year and hospital for inpatient treatment of PD and other basal ganglia disorders (ICD. G20–G23) grouped into quantitative classes for the year 2016.

	Cases	Proportion of Cases in 2016	Hospitals	Proportion of All Hospitals Which Treated PD Patients
Single Cases	490	1.0%	245	18.9%
<1 Case Monthly	3052	6.0%	495	38.2%
<1 Case Weekly	9076	17.9%	326	25.2%
1–10 Cases Weekly	28,590	56.4%	223	17.2%
>10 Cases Weekly	9503	18.7%	7	0.5%

**Table 6:** Distribution of inpatient treatment (ICD G20–G23) among the different departments for the year 2016.

ICD	Department of Neurology	Department of Internal Medicine	Department of Geriatrics	Other Departments
G20	76.9%	11.8%	7.3%	4.0%
G21	69.7%	17.9%	9.4%	3.0%
G23	84.3%	9.8%	4.0%	1.9%
Total	77.0%	12.0%	7.2%	3.8%

**Table 7:** Number of cases per year and hospitals for PD-MCT in PD (ICD G20–G23) grouped into classes for the year 2016.

	Cases	Proportion of Cases in 2016	Hospitals	Proportion of all Hospitals Which Treated PD Patients
Single Cases	31	0.3%	24	11.6%
<1 Case Monthly	304	2.6%	36	17.4%
<1 Case Weekly	2750	23.5%	97	46.9%
1–2 Cases Weekly	1759	15.0%	24	11.6%
>2 Cases Weekly	6878	58.7%	26	12.6%

**Table 8:** Distribution of PD-MCT (ICD G20+G21+G23 among the different departments for the year 2016.

OPS	Department of Neurology	Department of Internal Medicine	Department of Geriatric Medicine	Other Departments
8-97d.0	93.9%	5.1%	0.4%	0.6%
8-97d.1	94.3%	4.8%	0.8%	0.0%
8-97d.2	92.9%	6.7%	0.4%	0.0%
8-97d	94.1%	5.0%	0.7%	0.1%
